# Hepatitis B Virus, Hepatitis C Virus, and Human Immunodeficiency Virus Infection Among Premarital Screening Individuals in Saudi Arabia

**DOI:** 10.3389/ijph.2024.1607809

**Published:** 2024-08-26

**Authors:** Deemah S. Alfadhli, Suha M. Sulimani, Sahar M. Fadl, Ibtihal M. Bin Jumah, Abdullah F. Alanazi, Abdulaziz S. Alangari

**Affiliations:** ^1^ General Administration of Health Programs and Chronic Diseases, Ministry of Health, Riyadh, Saudi Arabia; ^2^ Department of Epidemiology and Biostatistics, College of Public Health and Health Informatics, King Saud bin Abdulaziz University for Health Sciences, Riyadh, Saudi Arabia; ^3^ King Abdullah International Medical Research Center, Riyadh, Saudi Arabia

**Keywords:** premarital, hepatitis B virus, hepatitis C virus, human immunodeficiency virus, prevalence

## Abstract

**Objective:**

Premarital screening is one of the most important strategies for preventing infectious diseases such as hepatitis B virus, hepatitis C virus, and human immunodeficiency virus in populations. This study aims to explore the prevalence of these viruses and their association with potential demographic factors among individuals undergoing premarital screening in Saudi Arabia.

**Methods:**

A cross-sectional study design using the National Healthy Marriage Program electronic registry in the Saudi Ministry of Health. Patients were selected from the premarital screening tests for the three blood-borne viruses. Data were obtained from January to August 2021 among 114,740 individuals.

**Results:**

Hepatitis B virus infection showed the highest prevalence followed by hepatitis C and human immunodeficiency viruses. Among those who were infected, men had higher infectious disease prevalence than women. The central and western regions had the highest percentages of infection.

**Conclusion:**

The studied infections pose a continuous public health issue among premarital screening individuals in Saudi Arabia. This study identified important demographic risk factors for these diseases and highlighted the need for future strategies and long-term plans at the national level.

## Introduction

Hepatitis B virus (HBV), hepatitis C virus (HCV), and human immunodeficiency virus (HIV) are ongoing worldwide health problems [1]. The contribution of acute viral hepatitis (A, B, C, and E) and HIV/AIDS according to the global burden of disease is well established in 1991, 2009, and 2019 global data [2]. Viral hepatitis is typically caused by a viral infection that affects the liver and its cells, resulting in liver inflammation and damage causing a variety of health issues. Among the five main types of hepatitis virus (A, B, C, D, and E), B and C, especially, result in chronic hepatitis in hundreds of millions of people and are the leading causes of cirrhosis, cancer, and deaths associated with viral hepatitis. According to the World Health Organization (WHO), 354 million people worldwide are infected with hepatitis B or C [3]. With vaccination, diagnostic tests, educational campaigns, and drugs, prevention is possible for some types of hepatitis. By 2030, the WHO aims to decrease the number of new cases by 90% and deaths by 65% [3]. Around 58 million people globally were infected with HCV, and approximately 290,000 people died [4]. Additionally, 296 million people were living with HBV infection, and an estimated 820,000 deaths occurred as a result; it is considered one of the leading causes of morbidity and mortality and a severe public health problem worldwide [5].

On the other hand, HIV is an infection that damages the immune system. The virus attacks and destroys the CD4 cells (white blood cells). This results in weakening the individual’s immunity against some opportunistic infections, such as bacterial, viral, fungal, and protozoal infections. Furthermore, thirty-nine million people worldwide were infected with HIV, 76% had access to antiretroviral therapy, and 14% of all people living with the virus without knowing they were infected [6]. In 2020, around 1.5 million people were living with HIV and around 680,000 contributed deaths. The WHO aims to reduce HIV infections to 335,000, and deaths to under 240,000 by 2030 [7]. It is evident that some of those who have HIV are co-infected with HCV or HBV. Around 10%–25% of HIV-infected people are co-infected with HBV, and approximately 30% are co-infected with HCV in the United States and Europe [8].

Although HBV, HCV, and HIV have distinct differences such as targeted organ systems, life cycles and host cell integration, they are somehow similar in the mode of transmission [9]. These viruses are considered blood-borne viruses, which can be transmitted through blood or body fluids (tears, saliva and sweat). For example, through sexual intercourse, needle sticks, and mother-to-child transmission (childbirth or breastfeeding) [10]. Raising awareness is key to preventing the health outcomes of these viruses that contribute to increased morbidity and mortality. In addition, early diagnosis and effective treatment of Sexually Transmitted Infections are important strategies to prevent these transmissions [11].

Twenty-six countries have adopted a premarital screening program to prevent these infectious diseases and some genetic disorders, including Saudi Arabia [12]. The Premarital Screening, or Healthy Marriage, Program is one of the essential programs in Saudi Arabia as well as other countries that adopted it [13]. This program limits the spread of genetic hemoglobin diseases, congenital anomalies, many psychological problems, and infectious diseases such as HBV, HCV, and HIV/AIDS. The program is part of a national project spearheaded by the Saudi Ministry of Health [14, 15]. The program started in 2004 as a program to help detect genetic disorders (sickle cell disease and thalassemia) and infectious diseases (HBV, HCV, and HIV) [14]. The screening program provides premarital examination alongside many other services, counseling sessions, and laboratory testing [16]. This study aims to explore the prevalence of HBV, HCV, and HIV and their association with potential demographic characteristics among individuals undergoing premarital screening through the Healthy Marriage Program in Saudi Arabia.

## Methods

### Study Design and Setting

**FIGURE 1:**
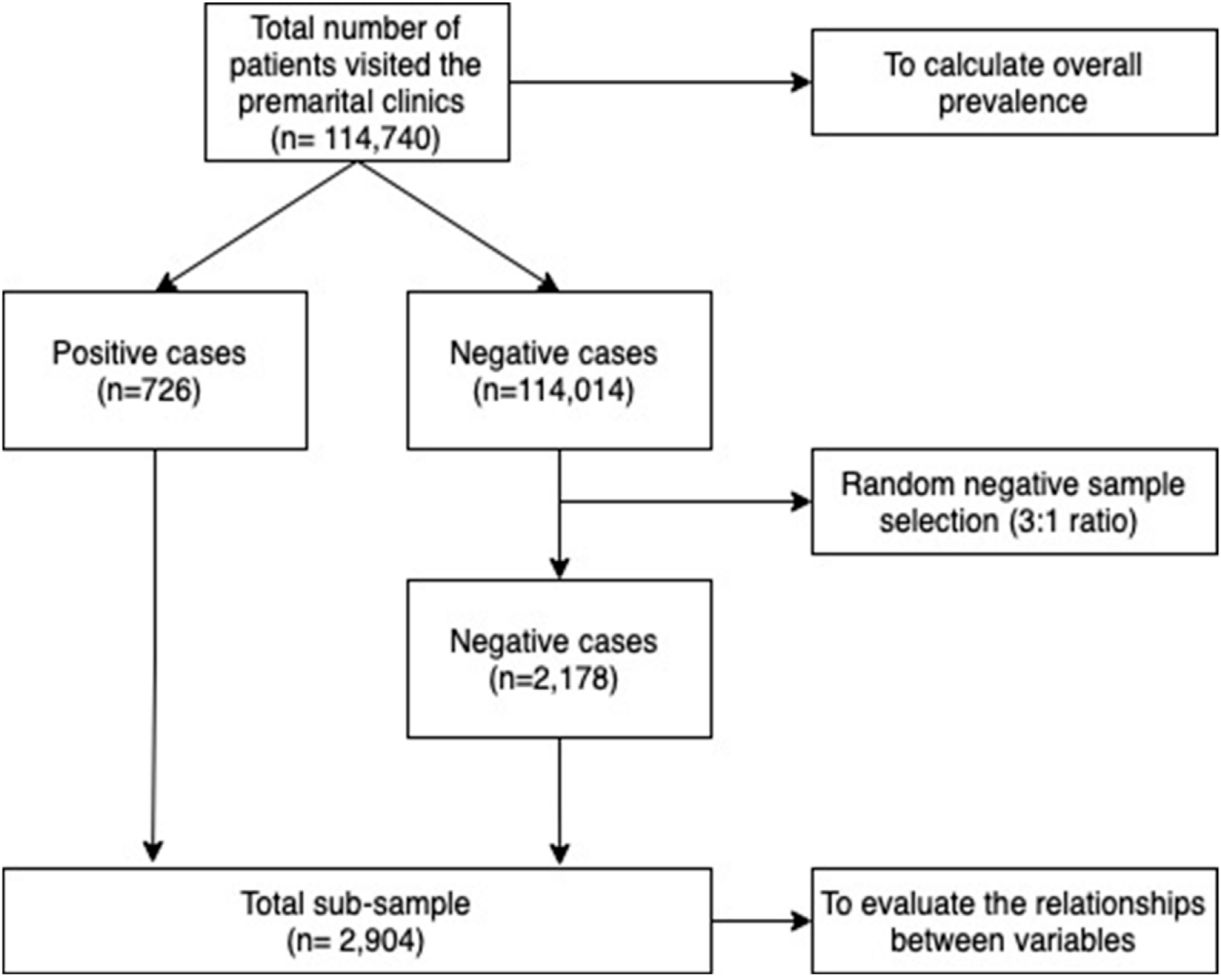
Sample flowchart (Saudi Arabia, 2021).

This was a cross-sectional study of 114,740 individuals in the premarital screening program of the Saudi Ministry of Health, conducted through the National Program Registry’s electronic database. The study was conducted from January to August 2021 in all 5 main regions of the Kingdom (Eastern, Southern, Northern, Western, and Central). It included 20 classified regions (Al-Baha, Al-Jawf, Al-Qassim, Asir, Dammam, Hail, Jizan, Al-Madinah, Makkah, Najran, Northern Borders, Riyadh, Tabuk, Al-Qurayat, Jeddah, Taif, Hafr Al-Batin, Al-Ahsa, Al-Qunfudhah, and Bisha). Since the premarital screening is mandatory for all individuals who are applying for a marriage certificate, all of those who got tested were included. Individuals under 18 years of age were excluded from the study. Controls were randomly chosen based on a 1:3 case-control method. There were 724 people infected in the sample. Of which, 2 cases with co-infection resulted in a total of n = 2178 controls (Figure 1). The Central Institutional Review Board (IRB) in the Saudi Ministry of Health reviewed and approved the study (Approval reference number: 21–81 M).

### Measures

Demographic variables were age (age at visit), gender (Male and Female), and region (Eastern, Southern, Northern, Western, and Central). Infectious diseases were diagnosed through a unified procedure, and healthcare centers must follow the Saudi Ministry of Health’s requirements to provide premarital screening services. Screening for HBV was done through the Hepatitis B surface antigen (HBsAg). If the test is positive, a confirmatory neutralization test is performed. A positive confirmatory neutralization test indicates an infection and no further tests are required. In the case of equivocal results, a Polymerase Chain Reaction (PCR) test is performed. Positive results indicate a confirmed infection. HCV is screened through the HCV antibody (Anti-HCV) test. If the results are positive, a confirmatory line immunoassay (LIA) test is performed. A positive or equivocal result mandates an HCV-PCR test, which determines the final infection status. For HIV screening, a fourth-generation HIV Ag/Ab test is performed. If the results are positive, a confirmatory LIA (Immunoblotting) test is performed. If equivocal results, an HIV-PCR test is performed, which determines the final infection status.

### Statistical Analysis

Descriptive statistics were used for the study variables, including frequencies and percentages. Logistic regression was used to evaluate associations between risk factors and the probability of infection. Odds ratios (OR) were calculated for unadjusted analyses (crude logistic regression) and significant variables were included in the adjusted analyses (multivariable logistic regression). Statistical significance was set at *p-value* < 0.05. The data were analyzed using IBM SPSS Statistics (Version 25.0) (IBM Corp., Armonk, NY, United States). The distribution map of infection prevalences among different provinces was produced by calculating rates per 100,000 visits among each province using QGIS Geographic Information System Software Version 3.28.1 (http://www.qgis.org).

## Results

The prevalence of any infection (HCV, HBV, or HIV) was 0.6% (630 cases per 100,000 visitors) among the total study population (n = 114,740). HBV showed the highest number of cases (600 cases, 523 cases per 100,000 visitors), followed by HIV (69 cases, 60 per 100,000 visitors) and HCV (57 cases, 50 per 100,000 visitors). Males were 50.9% of the sample and 55.7% in the sub-sample. More than half of the screened individuals fell within the age group 18–27 years (50.3% in the total sample and 55.7% in the sub-sample), followed by those in the 28 to 37 age group (36.0% in the total sample and 37.7% in the sub-sample). Central and western regions were the regions with the highest premarital screening visits, 28.4% and 28.1% respectively (Table 1).

**TABLE 1:** Sample characteristics (Saudi Arabia, 2021).

	All sample (n = 114,740)		Sub-sample (n = 2,904)	
Variable	n	(%)	n	(%)
Gender				
Male	58,438	(50.9)	1,618	(55.7)
Female	56,302	(49.1)	1,286	(44.3)
Age group				
18–27	57,688	(50.3)	1,223	(42.1)
28–37	41,330	(36.0)	1,095	(37.7)
38–47	9,994	(8.7)	348	(12.0)
≥48	5,728	(5.0)	238	(8.2)
Region				
Southern	19,390	(16.9)	497	(17.1)
Northern	11,012	(9.6)	253	(8.7)
Central	32,558	(28.4)	841	(29.0)
Eastern	19,484	(17.0)	466	(16.0)
Western	32,296	(28.1)	847	(29.2)
HIV				
Negative	114,671	(99.9)	2,835	(97.6)
Positive	69	(<0.1)	69	(2.4)
HCV				
Negative	114,683	(99.9)	2,847	(98.0)
Positive	57	(<0.1)	57	(2.0)
HBV				
Negative	114,140	(99.5)	2,304	(79.3)
Positive	600	(0.5)	600	(20.7)
Any infection				
Negative	114,016	(99.4)	2,180	(75.1)
Positive	724	(0.6)	724	(24.9)

The majority of infections were among the male gender with 71.3% among HBV cases, 77.2% among HCV cases, and 81.9% among HIV cases. The age group 28–37 was the most prevalent age with infection cases among all infection types. Among HBV cases, the age group 38–47 was the second most common age group (27.8%) followed by >48 years old (22.7%). As far as HCV infections, the age group >48 years old was the second most common age group with 26.3% followed by the age group 18–27 with 15.8%. HIV infections were most commonly prevalent among younger ages. The second most common age group was 38–47 with 15.9% followed by the age group 18–27 with 14.5%. The region distribution of positive infection cases was mostly in the central and western regions with the majority of cases in the western region (Table 2).

**TABLE 2:** Distribution of positive hepatitis B virus, hepatitis C virus, and human immunodeficiency virus cases by gender, age group, and region (n = 724) (Saudi Arabia, 2021).

	HBV (n = 600)			HCV (n = 57)			HIV (n = 69)		
	523 cases per 100,000 visitors			50 cases per 100,000 visitors			60 cases per 100,000 visitors		
	Cases		Cases per 100,000^a^	Cases		Cases per 100,000^a^	Cases		Cases per 100,000^a^
Variable	n	(%)	Rate	n	(%)	Rate	n	(%)	Rate
Gender									
Male	428	(71.3)	373	44	(77.2)	38	56	(81.2)	49
Female	172	(28.7)	150	13	(22.8)	11	13	(18.8)	11
Age group									
18–27	63	(10.5)	55	9	(15.8)	8	10	(14.5)	9
28–37	234	(39.0)	204	30	(52.6)	26	44	(63.8)	38
38–47	167	(27.8)	146	3	(5.3)	3	11	(15.9)	10
≥48	136	(22.7)	119	15	(26.3)	13	4	(5.8)	3
Region									
Southern	131	(21.8)	114	9	(15.8)	8	4	(5.8)	3
Northern	36	(6.1)	31	4	(7.0)	3	1	(1.4)	1
Central	173	(28.8)	151	11	(19.3)	10	19	(27.5)	17
Eastern	71	(11.8)	62	10	(17.5)	9	15	(21.7)	13
Western	189	(31.5)	165	23	(40.4)	20	30	(43.6)	26

HBV, Hepatitis B Virus; HCV, Hepatitis C Virus; HIV, Human Immunodeficiency Virus.

^a^ Rates were calculated among all visitors (n = 114,740).

As Table 3 depicts, the studied infectious diseases showed a significant association with age, gender, and region. Males are more likely to be infected with HBV (OR = 1.3; 95% CI: 1.1–1.7; *p* < 0.001), and HIV (OR = 2.8; 95% CI, 1.5–5.2; *p* < 0.001) than females. Older age categories showed an elevated probability of infection. Those who were >48 years old are more likely to be infected with HBV (OR = 21.0; 95% CI: 14.4–30.5; *p* < 0.001) and HCV (OR = 7.0; 95% CI, 2.9–16.7; *p* < 0.001) than 18–27 years old. Moreover, those who were aged 38–47 years old were more likely to be infected with HBV (OR = 15.9; 95% CI, 11.4–22.2; *p* < 0.001) and HIV (OR = 3.1; 95% CI, 1.3–7.4; *p* < 0.001) compared with 18–27 years old. In addition, those who were aged 28–37 years old were more likely to be infected with HBV (OR = 4.7; 95% CI, 3.5–6.3; *p* < 0.001), HCV (OR = 3.3; 95% CI, 1.5–7.0; *p* < 0.001) and HIV (OR = 4.0; 95% CI, 2.0–8.1; *p* < 0.001) compared with 18–27 years old. Those who live in the southern regions were more likely to get infected with HBV than the rest of the regions.

**TABLE 3:** Logistic regression analyses for factors associated with hepatitis B virus, hepatitis C virus, and human immunodeficiency virus infection (n = 2,904) (Saudi Arabia, 2021).

	HBV				HCV				HIV			
	AOR	95% CI		*p-value*	AOR	95% CI		*p-value*	AOR	95% CI		*p-value*
Gender				<0.001^a^				0.07				<0.001^a^
Female	1.0	Reference			1.0	Reference			1.0	Reference		
Male	1.3	1.1	1.7		1.8	0.9	3.5		2.8	1.5	5.2	
Age				<0.001^a^				<0.001^a^				<0.001^a^
18–27	1.0	Reference			1.0	Reference			1.0	Reference		
28–37	4.7	3.5	6.3		3.3	1.5	7.0		4.0	2.0	8.1	
38–47	15.9	11.4	22.2		1.0	0.3	3.8		3.1	1.3	7.4	
≥48	21.0	14.4	30.5		7.0	2.9	16.7		1.4	0.4	4.5	
Region				<0.001^a^		^b^				^b^		
Southern	1.0	Reference										
Northern	0.5	0.3	0.7									
Central	0.7	0.5	0.9									
Eastern	0.5	0.4	0.8									
Western	0.7	0.6	0.9									

AOR, Adjusted Odds Ratio; CI, Confidence Interval.

^a^ Significant test at *p*-value < 0.05.

^b^ There is not enough sample size to perform the test.

Models were adjusted for Gender, Age and Region. For HCV and HIV, region was excluded from the model.

**FIGURE 2:**
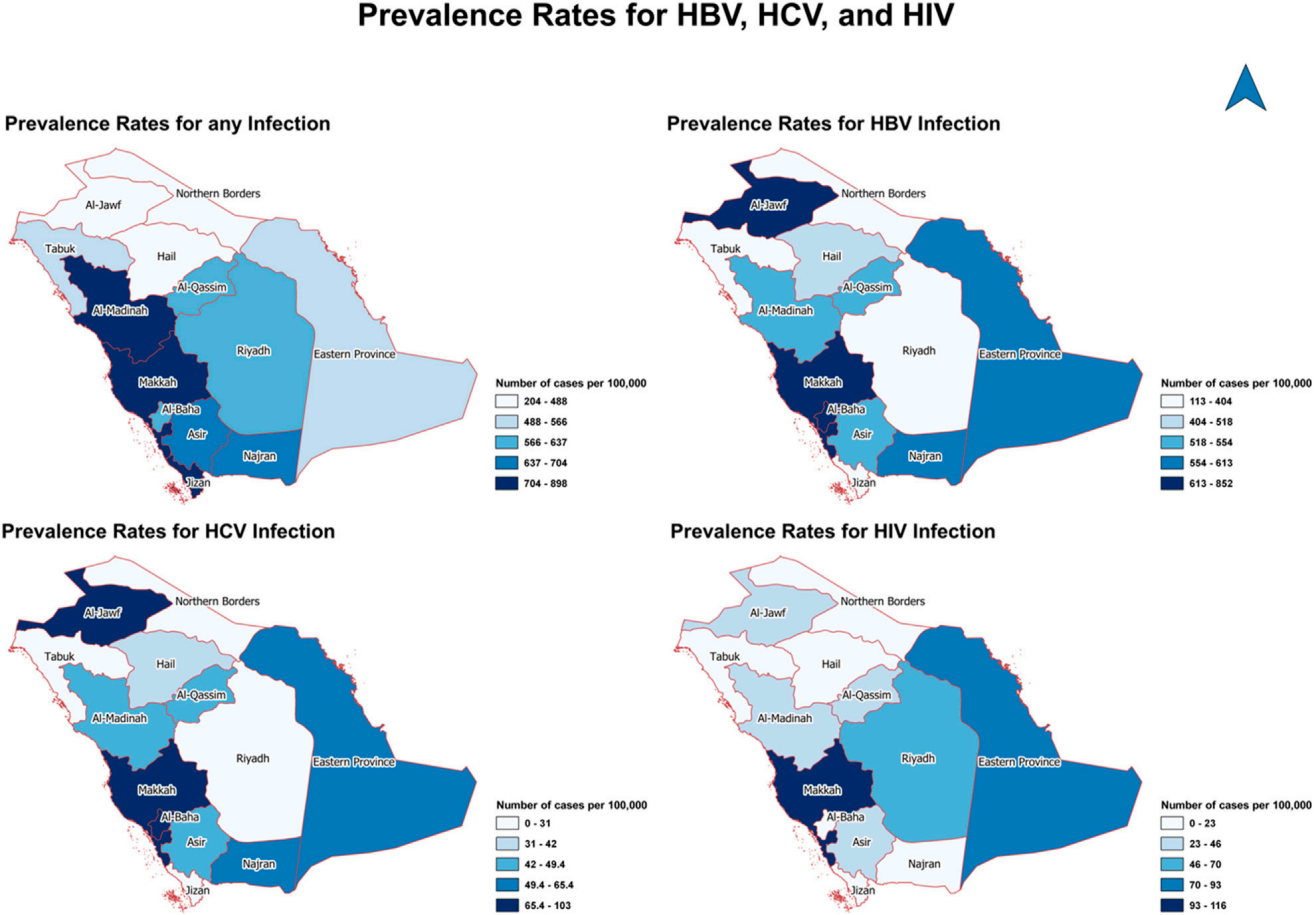
The geographical distribution of positive cases of hepatitis B virus, hepatitis C virus, and human immunodeficiency virus by provinces (Saudi Arabia, 2021).

Figure 2 shows the geographical distribution of the 724 positive cases out of 114,740 by provinces. Overall, Al-Madinah, Makkah, and Jizan were the most prevalent provinces with any type of infection (704–898 cases per 100,000). Similar distribution between HBV and HCV in Al-Jawf, Makkah, and Al-Baha since they were the most common areas for both infections (613–852 cases per 100,000 for HBV and 65–103 cases per 100,000). On the other hand, Makkah is the most prevalent province for HIV infection (93–116 cases per 100,000).

## Discussion

The current study reported that the prevalence of any of the infectious diseases studied among the visitors of the premarital screening clinics was 0.6%. HBV has the highest prevalence (0.5%) compared with other infectious diseases (<0.1 for both HCV and HIV). According to a previous study in Saudi Arabia, the prevalence of HBV and HCV were 0.52% and 0.05%, respectively, while the prevalence of HIV was 0.06% [17]. Recent results on infectious diseases in Mexico showed that HCV had a prevalence rate of 3.9%, while HBV showed 1.0% and HIV showed 2.9% prevalence [18]. A study in Tanzania showed that HIV had the highest prevalence, at 12.6%, compared with HBV and HCV (3.5%, and 3.5%, respectively) [19]. In Egypt, HBV and HCV tests were conducted on 3,420 samples from blood donors showing that 3.5% of donors reacted positively to HCV, and 1.4% reacted positively to HBV [20]. Moreover, a study in the Syrian Arab Republic where 3,168 individuals were chosen randomly out of 528 clusters, showed that the seroprevalence rate for HCV was 2.8% and for HBV was 5.6% in the northern and eastern parts of the region [21].

Gender, specifically being male, was an important factor in contracting infectious diseases among this sample. Men showed higher rates of HBV, HCV, and HIV infections compared with women. This finding is consistent with previous results. The overall prevalence of viral infections was higher in males (11.2%) compared with females (7.0%) [22]. Moreover, in 2018, premarital screening for viral hepatitis was performed involving 7,826 Saudis, and the prevalence of viral hepatitis was high among males (HBV, 1.9%, and HCV, 0.4%) compared with females (HBV, 1.43%, and HCV, 0.2%) [23]. In addition, a study conducted in 2011 found that infection in males was three times higher than in females [24]. On the other hand, according to previous findings in Saudi Arabia, there was no significant difference in infection between males compared with females [17].

Some differences in infection risk between age groups were observed. The age group 48 years and over has more likelihood of HBV and HCV infections. While the age group 28–37 years has more likelihood of HIV infection. On the other hand, the Al-Suwaidi and O’Brien study reported an increased risk of all three infectious diseases in those over 30 years of age. In addition, the results from the Jazan study showed a similar pattern as the age group 60 years and over had the highest prevalence of hepatitis B surface antigen (HBsAg) (22.4%) [22]. Furthermore, the prevalence was lowest for those under 20 years old, at only 2.5%. Although this study could not show any documented data to determine the reasons behind the increase in the number of cases, it showed a high number of positive cases in the following regions: Central, Western, and Eastern. In 2010, the regions with the highest positive results were Riyadh, Dammam, and Madinah, and the lowest cases were in Al-Qunfudhah [17]. This might be due to the population density in these areas as it increases the risk of such infections.

Our findings show only 2 patients with co-infection. A study by Amiri et al. in 2016, reported that the association between HIV and HBV in the general population produced a very low OR, close to zero percent (95% CI, 0.00–0.003) [25], while a study by Chen, Jia-Jia et al in 2011, reported an association between HBV and HIV (OR = 3.00; 95% CI, 1.90–4.73; *p* < 0.001), which is higher than our findings [24]. In contrast, national studies in the United States reported around 10% of people infected with HIV were also infected with HBV(8). Referred to as HIV/HBV co-infection, this type of infection might be primarily due to the sexual behaviors of men who have sex with men and injection drug users; however, because of the different cultures and traditions in Saudi Arabia, the explanations of the outcomes may differ between the two populations.

This study provides the most recent data related to the prevalence of HIV, HCV, and HBV infection for comparison with the results of previous studies and to emphasize the importance of screening programs in Saudi Arabia. With a sufficient national sample size (n = 114,740), the results will also help guide future studies to focus on specific populations, infections, or factors associated with infectious diseases. As the screening program’s goal is for individuals to have a safe and compatible marriage, individuals with positive screening results are required to obtain approval for the marriage to take place. They are also required to take the necessary treatment for life, provided free of charge by the Ministry of Health [16].

The limitations presented by the cross-sectional study design increase the chances for selection bias, in the inability to determine the actual number of patients with the disease between spouses. As the sample presented all persons who were intending to get married, we may have missed a substantial percentage of people who did not visit these clinics. Also, due to the small sample size of positive cases (n = 726), there is a potential for bias. In addition, we lacked information on medical history and other confounders, due to their unavailability in the database, making it difficult for us to determine the reasons for the high infection rates. Another factor to consider is the limited number of studies conducted in some regions; thus, results may not be comparable with some previous studies at the regional level.

### Conclusion

This research study showed the prevalence and important factors associated with HIV, HCV, and HBV infections, which may be considered during the development of premarital screening plans. The premarital examination program helps to prevent targeted diseases. However, patients’ unified medical records and databases lacked information about medical history and infectious diseases. In the future, we recommend that the Healthy Marriage Program collects the medical history including infectious diseases from patients to be added to the database, due to the need for studies to detect other risk factors and reduce the overall burden of HIV, HCV, and HBV infections with targeted interventions. This can be achieved by the development of new national measures and strategies as one of the priority issues in the country. On the other hand, due to the significant risk of infectious viruses, we need to focus on preventive plans for men attending the premarital examination program, emphasizing health education through outreach programs.
